# Osteopontin activates the diabetes-associated potassium channel TALK-1 in pancreatic β-cells

**DOI:** 10.1371/journal.pone.0175069

**Published:** 2017-04-12

**Authors:** Matthew T. Dickerson, Nicholas C. Vierra, Sarah C. Milian, Prasanna K. Dadi, David A. Jacobson

**Affiliations:** Department of Molecular Physiology and Biophysics, Vanderbilt University, Nashville, Tennessee, United States of America; Tel Aviv University Sackler Faculty of Medicine, ISRAEL

## Abstract

Glucose-stimulated insulin secretion (GSIS) relies on β-cell Ca^2+^ influx, which is modulated by the two-pore-domain K^+^ (K2P) channel, TALK-1. A gain-of-function polymorphism in *KCNK16*, the gene encoding TALK-1, increases risk for developing type-2 diabetes. While TALK-1 serves an important role in modulating GSIS, the regulatory mechanism(s) that control β-cell TALK-1 channels are unknown. Therefore, we employed a membrane-specific yeast two-hybrid (MYTH) assay to identify TALK-1-interacting proteins in human islets, which will assist in determining signaling modalities that modulate TALK-1 function. Twenty-one proteins from a human islet cDNA library interacted with TALK-1. Some of these interactions increased TALK-1 activity, including intracellular osteopontin (iOPN). Intracellular OPN is highly expressed in β-cells and is upregulated under pre-diabetic conditions to help maintain normal β-cell function; however, the functional role of iOPN in β-cells is poorly understood. We found that iOPN colocalized with TALK-1 in pancreatic sections and coimmunoprecipitated with human islet TALK-1 channels. As human β-cells express two K^+^ channel-forming variants of TALK-1, regulation of these TALK-1 variants by iOPN was assessed. At physiological voltages iOPN activated TALK-1 transcript variant 3 channels but not TALK-1 transcript variant 2 channels. Activation of TALK-1 channels by iOPN also hyperpolarized resting membrane potential (*V*_m_) in HEK293 cells and in primary mouse β-cells. Intracellular OPN was also knocked down in β-cells to test its effect on β-cell TALK-1 channel activity. Reducing β-cell iOPN significantly decreased TALK-1 K^+^ currents and increased glucose-stimulated Ca^2+^ influx. Importantly, iOPN did not affect the function of other K2P channels or alter Ca^2+^ influx into TALK-1 deficient β-cells. These results reveal the first protein interactions with the TALK-1 channel and found that an interaction with iOPN increased β-cell TALK-1 K^+^ currents. The TALK-1/iOPN complex caused *V*_m_ hyperpolarization and reduced β-cell glucose-stimulated Ca^2+^ influx, which is predicted to inhibit GSIS.

## Introduction

Glucose-stimulated insulin secretion (GSIS) from pancreatic β-cells is critical to the maintenance of glucose homeostasis. This process requires pancreatic β-cell Ca^2+^ influx through voltage dependent calcium channels (VDCCs), which are activated by plasma membrane depolarization. Two-pore-domain potassium (K2P) channels are important to this process due to their role in regulating beta-cell membrane potential (*V*_m_) [1–3]. For example, the TWIK-related alkaline pH-activated K2P (TALK)-1 channel and the TWIK-related acid-sensitive K2P (TASK)-1 channel hyperpolarize β-cell *V*_m_ [4–6]. These K2P channels help to set the basal beta-cell *V*_m_ with their continuous K^+^ flux at all physiological *V*_m_ [7–9]. However, the conductance of K2P channels is significantly less than that of the ATP sensitive K^+^ channel (K_ATP_), the primary K^+^ channel responsible for setting the beta-cell *V*_m_ under low glucose conditions [8, 10, 11]. Thus, the activity of beta-cell K2P channels would be expected to play a more prominent role in controlling the *V*_m_ during glucose stimulation and K_ATP_ inhibition. Moreover, the small conductance of beta-cell K2P channels stabilizes the glucose induced plateau potential from which action potentials fire at a *V*_m_ that is optimal for activation of VDCCs [12, 13]. While K2P channels play an important role in regulating beta-cell *V*_m_ and Ca^2+^ influx, the mechanism(s) controlling beta-cell K2P channels have not been elucidated.

Transcriptome analysis from many independent studies has shown that *KCNK16*, which codes for the TALK-1 channel, is the most abundant K^+^ channel transcript found in human β-cells [14–18]. Moreover, *KCNK16* transcript is predominantly expressed in pancreatic islets and only observed via northern blot analysis in human pancreatic tissue [7, 8]. There are four human TALK-1 transcript variants, including two that form functional K^+^ channels (TALK-1a (transcript variant 2 (T2)) and TALK1-b (transcript variant 3 (T3))) [8, 9]. We have shown that functional TALK-1 K^+^ channels are produced in mouse and human β-cells where they tune β-cell electrical excitability by polarizing β-cell *V*_m_. Islet β-cells from TALK-1 knockout (KO) mice exhibit increased *V*_m_ depolarization, augmented Ca^2+^ influx, and elevated second phase GSIS [6]. In addition, a nonsynonymous polymorphism in TALK-1 (rs1535500) that results in the substitution of an alanine (A) at position 277 with a glutamate (E) has been linked to an increased risk of type 2 diabetes [19, 20]. This polymorphism results in a gain-of-function (GOF) of TALK-1 channels increasing their open probability, which is predicted to hyperpolarize β-cell *V*_m_ and decrease Ca^2+^ influx and insulin secretion [6]. Although TALK-1 plays a substantial role in β-cell function, mechanisms that control TALK-1 channel activity in β-cells have not been examined.

Similar to several other K2P channels, TALK-1 is pH sensitive, with increased activity under alkaline conditions and lower activity under acidic conditions [21, 22]. However, the channel is not completely inhibited under acidic conditions and exhibits K^+^ conductance across the entire physiological pH range islets are exposed to. Interestingly, TALK-1 channels are also activated by singlet oxygen and nitric oxide (NO) [21]. It is important to note that the factors shown to regulate TALK-1 function have only been demonstrated on heterologously expressed TALK-1 channels. Therefore, the work detailed here is the first to examine a regulator of TALK-1 channel activity in primary β-cells.

Here we identified a number of membrane-associated and cytosolic proteins expressed in pancreatic islets that interact with TALK-1 using a powerful technique known as a split-ubiquitin based Membrane Yeast Two-Hybrid (MYTH) assay, which has been used to reveal the pancreatic islet glucagon-like peptide 1 receptor (GLP-1R) interactome [23–26]. We found that a subset of TALK-1-interacting proteins modulated K^+^ channel currents. From this group intracellular osteopontin (iOPN) displayed the most pronounced effect on TALK-1 activity. We further showed that the interaction between TALK-1 and iOPN occurs in mouse and human beta-cells. Moreover, this interaction activated mouse beta-cell K2P currents, which were reduced by iOPN knockdown (KD) in wild type but not in TALK-1 deficient beta-cells. Finally, we found that activation of TALK-1 by iOPN hyperpolarized the *V*_m_ and reduced beta-cell glucose-stimulated Ca^2+^ influx. These studies reveal the first beta-cell TALK-1 channel interactome and find that one of these novel interactions with iOPN tunes TALK-1 K^+^ flux to modulate Ca^2+^ entry.

## Materials and methods

### Split ubiquitin-based Membrane Yeast Two-Hybrid (MYTH) assay

Healthy human islets were provided through an approved protocol by the NIH supported Integrated Islet Distribution Program (IIDP) (https://iidp.coh.org/). Written consent was obtained for deceased donors by the IIDP in accordance with guidelines set forth by the NIH prior to receiving human islets for our studies. All work detailed here was approved by the Vanderbilt University Health Sciences Committee Institutional Review Board (IRB# 110164). The studies presented here do not include human subjects; there was no interaction with the donors and there was no identifiable private information associated with the human islets provided by the IIDP. RNA was isolated and reverse transcriptase polymerase chain reaction (RT-PCR) was used to generate a human islet cDNA library as previously described [5]. A split ubiquitin system of plasmids were prepared comprised of fusion proteins of the islet cDNA library and N-terminal half of the ubiquitin moiety (NubG) along with a plasmid expressing a fusion protein of TALK-1 transcript variant 3 (T3) (accession: NM_001135106.1) and the C-terminal half of the ubiquitin moiety (Cub) according to the manufacturer instructions (Dualsystems Biotech). A MYTH screen was conducted according to the manufacturer instructions (Dualsystems Biotech) to identify proteins that interact with TALK-1 T3.

### Coimmunoprecipitation of TALK-1-FLAG and iOPN-V5 from human cells (HEK293)

HEK293 cells were grown to 90% confluence in 100 mm tissue culture dishes in Dulbecco’s Modified Eagle Media (DMEM) GlutaMax-I (Thermo Fisher Scientific) supplemented with 10% fetal bovine serum (FBS, Gibco), 100 IU∙ml^−1^ penicillin (Gibco), and 100 mg∙ml^−1^ streptomycin (Gibco) (complete media (CM)) at 37°C, 5% CO_2_. Cells were transfected for 48 hrs with 7.5 μg of pcDNA3.1 V5-His-TOPO expressing iOPN-V5 (NM_J04765.1) and 7.5 μg of pcDNA3.1 expressing TALK-1 T3-FLAG, TALK-1 T3 A277E (rs1535500)-FLAG, or TALK-1 transcript variant 2 (T2) (NM_032115.3)-FLAG using 22.5 μL Lipofectamine 3000 and 30 μL P3000 in a total volume of 500 μL of serum and antibiotic-free DMEM GlutaMax-I. Cells were washed twice with 10 mL of 1x phosphate buffered saline (PBS) and gently scrapped into 1 mL of radioimmunoprecipitation (RIPA) buffer on ice. The cell suspensions were dispersed by pipetting and sonicated on ice six times in three second pulses with a sonic dismembrator model 100 (Thermo Fisher Scientific). Lysates were incubated at 4°C for 30 min on a rotating rack then centrifuged for 10 min at 12,000 RPM, 4°C. The clarified lysates were transferred to clean microcentrifuge tubes and incubated 1 hr with 5 μg of mouse anti-FLAG M2 antibody (Sigma Aldrich). 40 μL of protein A/G magnetic beads (Pierce) per lysate sample were washed three times with RIPA buffer then the mixture of lysates and anti-FLAG antibodies were added to the magnetic beads and incubated overnight at 4°C on a rotating rack. Lysates were removed from the magnetic beads and saved for later analysis. The magnetic beads were washed three times with RIPA buffer and proteins were eluted for 10 min at room temperature with 100 μg/mL of FLAG peptide (Sigma Aldrich). The protein samples were stored at -20°C until analyzed. The samples were thawed on ice, combined 1:1 with 2x Laemmli buffer (Bio-Rad), and incubated 10 min at 95°C. The protein samples were cooled to room temperature and 40 μL of each were resolved on a NuPAGE 4–12% Bis-Tris Gel (Life Sciences) at 100 mV for 2 hrs. The proteins were transferred to a nitrocellulose membrane for 90 min at 100 mV on ice. The membrane was blocked in 3% blot-grade blocker (Bio-Rad) in 1x PBS (blocking solution) for 2 hrs at room temperature. The membrane was then incubated overnight with 1:500 rabbit anti-V5 (Bethyl Labs) in blocking solution. The membrane was washed three times (5 min per wash) in 1x PBS with 0.5% Tween 20 (Sigma Aldrich) and incubated 2 hrs with 1:2500 donkey anti-rabbit HRP-conjugated secondary (Jackson ImmunoResearch Laboratories) in blocking solution with 0.5% Tween 20. The membrane was washed three times (5 min per wash) in 1x PBS with 0.5% Tween 20 and once (5 min) in 1x PBS. V5 tagged protein bands were visualized using SuperSignal™ West Pico Chemiluminescent Substrate (Thermo Fisher Scientific). After imaging the membrane was washed for 10 min in diH_2_O, incubated in 0.2 M NaOH for 10 min, and washed for 10 min in diH_2_O. It was then blocked as detailed above, incubated overnight with 1:500 mouse anti-FLAG (Sigma Aldrich), washed as above, incubated 2 hrs with 1:2500 donkey anti-mouse HRP-conjugated secondary (Invitrogen) in blocking solution with 0.5% Tween 20, washed as above, and FLAG tagged protein bands visualized as indicated previously.

### Coimmunoprecipitation of TALK-1 and iOPN from primary human islets

Human islets were provided by the IIDP and upon arrival 5000 islet equivalents (IEQs) were handpicked and pooled. The islets were washed once with cold PBS, resuspended in 1 mL of RIPA buffer, and sonicated on ice using a sonic dismembrator in 3 sec pulses three times. TALK-1 and iOPN were coimmunoprecipitated as detailed in the previous section using 5 μg of rabbit anti-TALK-1 (Novus Biologicals). Proteins were resolved on a 4–12% Bis-Tris Gel and transferred to a nitrocellulose membrane. The membrane was blocked as above, incubated with 1:500 mouse anti-iOPN (Santa Cruz) overnight at 4°C, washed three times with in 1x PBS with 0.5% Tween 20, incubated for 2 hrs with 1:2500 donkey anti-mouse HRP-conjugated secondary, again washed three times with 1x PBS with 0.5% Tween 20, and iOPN protein was visualized with SuperSignal™ West Femto Maximum Sensitivity Substrate (Thermo Fisher Scientific). The membrane was stripped and reprobed for TALK-1 with 1:500 rabbit anti-TALK-1 and 1:2500 donkey anti-rabbit HRP-conjugated secondary.

### TALK-1 and iOPN immunofluorescent analysis

HEK293 cells were grown to 90% confluence in 35 mm tissue culture dishes in CM at 37°C, 5% CO_2_. Cells were transfected for 24 hrs with 1.5 μg of pcDNA3.1 V5-His-TOPO encoding iOPN-V5 and 1.5 μg of pcDNA3.1 encoding TALK-1 T3-FLAG or pcDNA3.1 expressing EGFP using 3.75 μL Lipofectamine 3000 and 5 μL P3000 in a total volume of 250 μL of unsupplemented DMEM GlutaMax-I. Glass slide covers were coated with 50 μg/mL of poly-D-lysine (Sigma Aldrich) for 2 hrs at room temperature, washed twice with diH_2_O, and air dried. The transfected cells were washed with 300 μL versene (Invitrogen) and trypsinized with 300 μL 0.05% trypsin-EDTA (Invitrogen). The trypsin was neutralized by adding 700 μL DMEM GlutaMax-I supplemented with 1% FBS and the cells plated on the poly-D-lysine coated glass slide covers as single cells and incubated overnight in CM at 37°C, 5% CO_2_. The cell coated cover slips were either washed with 1x PBS then blocked while still viable for 30 min on ice in 1x PBS with 0.2% BSA and 2% normal donkey serum (NDS, Jackson ImmunoResearch Laboratories) or fixed for 10 min with 4% paraformaldehyde and washed with 1x PBS prior to blocking. The cells were then incubated 2 hrs on ice with 1:200 rabbit anti-TALK-1 and 1:200 goat anti-V5 in 1x PBS with 0.2% BSA and 1% NDS. All cells were incubated 10 min with 4% paraformaldehyde, washed three times with 1x PBS, then incubated 1 hr at room temperature with 1:300 anti-goat Cy5 antibody and 1:300 anti-rabbit Alexafluor 488 (Jackson ImmunoResearch Laboratories). Cells were washed three times with 1x PBS and mounted on glass slides using 15 μL SlowFade Gold with DAPI (Life Technologies). The samples were cured overnight at room temperature protected from light then stored at 4°C until analyzed. Fluorescence imaging was performed using a Nikon Eclipse TE2000-U microscope equipped with an epifluorescence illuminator (Sutter Instrument Company), a CCD camera (HQ2; Photometrics, Inc), and Nikon Elements software (Nikon, Inc). Colocalization analysis of TALK-1 and iOPN was carried out using the ImageJ Fiji image processing pack. Mander’s coefficients (tM1 and tM2) were calculated as indicators of TALK-1 colocalization with iOPN (tM1) and iOPN colocalization with TALK-1 (tM2) respectively where a value of 1.0 corresponds to complete colocalization and -1.0 corresponds to no colocalization.

Fixed, paraffin-embedded pancreas tissue from an adult human donor (female, Caucasian, 79) was provided by the NCI funded Cooperative Human Tissue Network (CHTN) (https://www.chtn.org/). Written consent was obtained for deceased donors by the CHTN in accordance with IRB guidelines prior to reception of human pancreatic tissue by our lab. As outlined above, all work here was in compliance with IRB guidelines (IRB# 110164). Pancreas sections (5-mm) were prepared, rehydrated, and subjected to antigen retrieval with a citrate buffer according to manufacturer instructions (Vector Laboratories, Inc.). The slices were blocked for 2 hr in 1x PBS with 0.2% BSA, 2% NDS, and 0.05% Triton X-100 (Sigma Aldrich) and incubated overnight at 4°C with 1:175 rabbit anti-TALK-1 (Sigma) and 1:100 mouse anti-iOPN (MPIIIB10) (Developmental Studies Hybridoma Bank (DSHB)) in 1x PBS with 0.2% BSA, 1% NDS, and 0.05% Triton X-100. The slices were washed three times with PBS for 5 min and incubated 2 hrs protected from light at room temperature with anti-rabbit Cy3 (Invitrogen) and 1:500 anti-mouse Alexafluor 488 (Invitrogen) in 1x PBS with 0.2% BSA, 1% NDS, and 0.05% Triton X-100. Nuclei were stained using Prolong Gold mountant with DAPI and imaged with an Eclipse TE2000-U microscope. TALK-1 and iOPN colocalization was quantified by calculating tM1 and tM2 as detailed above.

### Mouse islet and β-cell isolation and culture

Wild type (WT) and TALK-1 knockout (KO) islets were isolated as previously described from the pancreata of 7- to 10-week old mice [27]. All experiments herein and mice used in this work were handled in compliance with protocols that have been approved by the Vanderbilt University Animal Care and Use Committee (protocol # M1600063-00). The islets were dispersed into single cells by pipetting in 0.0075% trypsin-EDTA to and cultured 18 hrs in poly-d-lysine coated 35 mm glass-bottom dishes (Cellvis) in RPMI 1640 supplemented with 15% FBS, 100 IU∙ml^−1^ penicillin, and 100 mg∙ml^−1^ streptomycin (islet media) at 37°C, 5% CO_2_. Immediately after plating, the cells were transfected using 1 μL Lipofectamine 3000 and 1 μL P3000 in a total volume of 15 μL of unsupplemented DMEM GlutaMax-I with 400 ng of a pLL3.7 vector (Addgene) containing the coding sequence for OPN shRNA (adapted from Wai et al.) and an EGFP reporter or a control pLL3.7 vector containing the coding sequence for the EGFP reporter [28]. The cells were maintained in islet media at 37°C, 5% CO_2_ and used for testing between 48 and 72 hrs. The efficiency of iOPN knockdown was also determined by immunofluorescent analysis. Osteopontin shRNA was expressed in dispersed β-cells for 48 hrs then the cells were fixed with 4% paraformaldehyde (PFA) for 40 min on ice. The cells were blocked for 1 hr in PBS with 0.1% Tween 20, 1% BSA, and 10% NDS then incubated overnight at 4°C with 1:1000 rabbit anti-OPN (ab8448) (Abcam) in the same solution. The cells were washed with PBS three times for ten minutes each time then incubated 1 hr at room temperature with 1:1000 donkey anti-rabbit Alexa Fluor 546 (Thermo Fisher Scientific) in PBS with 0.1% Tween 20, 1% BSA, and 2% NDS. The cells were again washed with PBS three times for ten minutes each time. Fluorescence imaging was performed using a Zeiss LSM710 META inverted confocal microscope with Zeiss ZEN microscope software. The average OPN immunofluorescence intensity of β-cells expressing EGFP was compared to β-cells without EGFP as an indicator of iOPN knockdown using the ImageJ Fiji image processing pack.

### Whole cell voltage clamp recordings

Stable cell lines with tetracycline inducible TALK-1 T2 (T2H16), TALK-1 T3 (T3H16), or TALK-1 T3 A277E (T3H16 A277E) expression were prepared using a pcDNA5/TO mammalian expression vector (Thermo Fisher Scientific) containing the coding sequence of TALK-1 T3 in T-REx-293 cells (Thermo Fisher Scientific). T3H16 cells were grown to 90% confluence in 35 mm tissue culture dishes in CM at 37°C, 5% CO_2_. Cells were transfected for 24 hrs with 2.625 μg of pcDNA3.1 V5-His-TOPO expressing iOPN-V5 or pcDNA3.1 expressing pyruvate kinase M2 (PKM2) as a noninteracting control and 0.375 μg of pcDNA3.1 expressing pEGFP-N1 using 3.75 μL Lipofectamine 3000 and 5 μL P3000 in a total volume of 250 μL of unsupplemented DMEM GlutaMax-I. The following day, the transfected cells were washed with 300 μL versene and trypsinized with 300 μL 0.05% trypsin-EDTA. The trypsin was neutralized by adding 700 μL CM and the cells dispersed by pipetting. Single cells were seeded in 35 mm glass-bottom dishes in CM supplemented with 1 μg/mL tetracycline (Sigma Aldrich) and incubated overnight in CM at 37°C, 5% CO_2_. A whole-cell patch clamp technique was employed to record TALK-1 channel currents in single cells using an Axopatch 200B amplifier with pCLAMP10 software (Molecular Devices). Cells were washed twice with HEK293 extracellular buffer (EC) with (in mmol/L) 140.0 NaCl, 2.0 CaCl_2_, 5.0 KCl, 10.0 HEPES, 2.0 MgCl_2_, 1.2 KH_2_PO_4_, and 11.0 glucose, adjusted to pH 7.4 with NaOH. During recording, samples were perfused with HEK EC without CaCl_2_ (adjusted to pH 7.4 with NaOH). Patch electrodes (6–12 MΩ) were filled with HEK293 intracellular solution (IC) with (in mmol/L) 10.0 NaCl, 1.0 CaCl_2_, 140.0 KCl, 1.0 MgCl_2_, 0.1 EGTA, 10.0 HEPES, and 8.0 Mg-ATP (adjusted to pH 7.2 with KOH).

Alternatively, transfected (as determined by EGFP fluorescence) WT and TALK-1 KO mouse β-cells were analyzed using the same whole-cell patch clamp technique. The cells were washed twice with Krebs-Ringer–HEPES buffer (KRHB) with (in mmol/L) 119.0 NaCl, 2.0 CaCl_2_, 4.7 KCl, 25.0 HEPES, 1.2 MgSO_4_, 1.2 KH_2_PO_4_ (adjusted to pH 7.4 with NaOH), and supplemented with 11.0 mM glucose. During recording, samples were perifused with KRHB without CaCl_2_ and with (in mmol/L) 0.2 tolbutamide (MP Biomedicals), 10.0 tetraethylammonium chloride hydrate (TEA, Thermo Fisher Scientific), and 1.0 ethylene glycol-bis(β-aminoethyl ether)-N,N,N',N'-tetraacetic acid (EGTA, Sigma Aldrich) (adjusted to pH 7.4 with NaOH). Patch electrodes (6–12 MΩ) were filled with islet cell IC with (in mmol/L) 140.0 KCl, 1.0 MgCl_2_, 10.0 EGTA, 10.0 HEPES, and 8.0 Mg-ATP (adjusted to pH 7.2 with KOH).

### Plasma membrane potential recordings

T3H16 cells were grown to 90% confluence in 35 mm tissue culture dishes in CM at 37°C, 5% CO_2_. Cells were transfected for 24 hrs with 2.625 μg pcDNA3.1 V5-His-TOPO expressing iOPN-V5 or 2.625 μg pcDNA3.1 expressing PKM2 and 0.375 μg pcDNA3.1 expressing pEGFP-N1 using 3.75 μL Lipofectamine 3000 and 5 μL P3000 in a total volume of 250 μL of unsupplemented DMEM GlutaMax-I. HEK293 cells were transfected in an identical manner with iOPN or PKM2 expressing plasmid along with pEGFP-N1 expressing plasmid. HEK293 cells were employed in place of uninduced T3H16 cells in order to prevent the influence of low level background TALK-1 channel expression present in the T3H16 cell line. The following day, the transfected cells were washed with 300 μL versene and trypsinized with 300 μL 0.05% trypsin-EDTA. The trypsin was neutralized by adding 700 μL CM and the cells dispersed by pipetting. Single cells were plated in 35 mm glass-bottom dishes in CM supplemented with 1 μg/mL tetracycline and incubated overnight in CM at 37°C, 5% CO_2_. A perforated patch clamp technique was employed to record *V*_m_ in single cells expressing TALK-1 T3 with iOPN or PKM2 or iOPN or PKM2 alone using an Axopatch 200B amplifier with pCLAMP10 software. Cells were washed twice with HEK293 EC then recorded in HEK293 EC. Patch electrodes (4–6 MΩ) were filled with HEK293 IC with (in mmol/L) 10.0 NaCl, 1.0 CaCl_2_, 140.0 KCl, 1.0 MgCl_2_, 0.1 EGTA, and 10.0 HEPES (adjusted to pH 7.2 with KOH) supplemented with 20 μg/mL amphotericin B.

The *V*_m_ of transfected WT and TALK-1 KO mouse β-cells was also measured using the same patch clamp technique employed for HEK293 cell recording. Mouse islets were isolated, dispersed into cell clusters of 10–20 cells, transfected with OPN shRNA/EGFP plasmid or EGFP plasmid alone, cultured on poly-d-lysine coated glass dishes, and recorded between 48 and 72 hrs post transfection. A perforated patch clamp technique was employed to record *V*_m_ in transfected cells using an Axopatch 200B amplifier with pCLAMP10 software. Cells were washed twice with KRHB with 11.0 mM glucose then *V*_m_ recording was started while perifusing fresh KRHB with 11.0 mM glucose. After 5–10 min of recording, perifusion was switched to KRHB with 2 mM glucose and *V*_m_ monitored until electrical activity ceased and *V*_m_ became hyperpolarized for at least 3 min. Both β-cell resting *V*_m_ (at 2 mM glucose) and plateau *V*_m_ (from which action potentials fire) were monitored. (Patch electrodes (4–6 MΩ) were filled with islet cell IC with (in mmol/L) 140.0 KCl, 1.0 MgCl_2_, and 5.0 HEPES (adjusted to pH 7.2 with KOH) supplemented with 20 μg/mL amphotericin B.

### Glucose-stimulated Ca^2+^ influx Fura-2 AM imaging

Mouse islet clusters were prepared and transfected with OPN shRNA or an empty control plasmid as outlined in the previous section. The cells were loaded with 2 μM Fura-2-acetoxymethyl ester (AM) (Thermo Fisher Scientific) for 25 min at 37°C, 5% CO_2_, washed twice with KRHB with 2.0 mM glucose, and incubated 20 min in KRHB with 2.0 mM glucose at 37°C, 5% CO_2_. Fluorescence imaging was performed using a Nikon Eclipse TE2000-U microscope and the data was analyzed using Nikon Elements software. Cells were perifused at 37°C with a flow of 2 mL/min KRHB with 2.0 mM glucose for 2 min then perifused under identical conditions with KRHB with 11.0 mM glucose for 20 min. The ratios of emitted fluorescence intensities at excitation wavelengths of 340 and 380 nm (F_340_/F_380_) were recorded every 5 seconds.

### Statistical analysis

Experimental data are presented as mean values ± SEM. Unpaired two-tailed t-tests and paired two-tailed t-tests were used to determine statistical significance between test groups as appropriate. P < 0.05 was considered statistically significant.

## Results

### Identification of human islet TALK-1-interacting proteins using a MYTH assay

To identify TALK-1-interacting proteins (Table 1), a human pancreatic islet cDNA library was generated and screened in a MYTH assay with TALK-1 T3 as a bait. The MYTH assay was run under stringent conditions (with a concentration of 15 mM 3-aminotrizole (3-AT)) to eliminate false positives. To confirm that colonies arising from this assay represented direct interactions with TALK-1, the prey cDNA was retransformed into yeast containing TALK-1 and only the hits that grew successfully with selective media were further evaluated. The strength of TALK-1 interactions was determined via a colorimetric HTX- galactosidase assay and the most robust interactions chosen for further testing. In total we identified 21 proteins from pancreatic islets with diverse functions that interacted with TALK-1 T3 (Table 1).

**Table 1:** Summary of human islet TALK-1-interacting proteins.

Gene Accession #	Protein	Localization; Known Functions
ARL61P5 NM_006407.3	ADP Ribosylation Factor Like GTPase 6 Interacting Protein 5	ER, extracellular, plasma membrane, cytoskeleton; Inhibits SLC1A1/EAAC1 glutamate transport in a PKC activity-dependent manner, may be involved in membrane traffic
CCL2 NM_002982.3	C-C Motif Chemokine Ligand 2	Extracellular, ER, cytosol, plasma membrane; Chemotactic for monocytes/basophils, Binds to chemokine receptors CCR2/4
CD63 NM_001780.5	CD63 Molecule	Extracellular, plasma membrane; Roles in cell differentiation/activation/growth/motility
CD9 NM_001330312.1	CD9 Molecule	Extracellular, plasma membrane; Roles in cell differentiation/adhesion/signal transduction
CLDN10B NM_006984.4	Claudin 10b	Plasma membrane; Regulates paracellular permeability, selective for cations
CNIH1 NM_005776.2	Cornichon Family AMPAR Auxiliary 1	ER, Golgi; Cargo receptor for COPII-mediated vesicular transport from ER to Golgi
DNER NM_139072.3	Delta/notch like EGF repeat containing	Plasma membrane, extracellular; Regulation of the Notch signaling pathway
ITGB1 BC020057.1	Integrin, β1	Extracellular, plasma membrane, cytoskeleton, cytosol, ER; Subunit of the fibronectin receptor
MKNK2 NM_199054.2	MAPK Interacting Ser/Thr Kinase 2	Nucleus, cytosol; CAMK Ser/Thr protein kinase, activated by MAPK, phosphorylates eIF4E
PDE8A NM_001243137.1	Phosphodiesterase 8A	Cytosol, extracellular; Modulation of cellular processes by regulation of secondary messenger cAMP
PERP NM_022121.4	TP53 Apoptosis Effector	Plasma membrane, mitochondrion, Golgi; Promoter of p53-dependent apoptosis, direct translational target of p53
REEP3 NM_001001330.2	Receptor Accessory Protein 3	ER, cytoskeleton; Links ER to microtubules during cell division, nuclear envelope reassembly after mitosis
SELK NM_021237.3	Selenoprotein K	ER; Forms a complex with DHHC6, required for IP3R palmitolyation
SPP1 NM_001040058.1	Osteopontin	Extracellular, cytosol, plasma membrane; Immune response, bone remodeling, anti-apoptosis
SSR3 NM_007107.4	Signal Sequence Receptor, gamma	ER, plasma membrane, cytosol; Protein translocation across ER membrane
SYNGR4 NM_012451.3	Synaptogyrin 4	Plasma membrane; Unknown
TM4SF4 NM_004617.3	Transmembrane 4 L Six Family Member 4	Plasma membrane; Cell proliferation and signal transduction
TMEM14A NM_014051.3	Transmembrane Protein 14A	Plasma membrane, extracellular, ER, mitochondrion; Modulates mitochondrial membrane potential
TMEM217 NM_001162900.1	Transmembrane Protein 217	Plasma membrane; Unknown
TMX2 NM_015959.3	Thioredoxin related TM protein 2	ER, cytosol, mitochondrion, plasma membrane; Palmitoylated TMX2 is targeted to the MAM
TSPAN8 NM_004616.2	Tetraspanin 8	Extracellular, plasma membrane, ER; Roles in cell differentiation/activation/growth/motility

### Protein interactions identified by MYTH assay also occur in human cells (HEK293)

**Fig 1:**
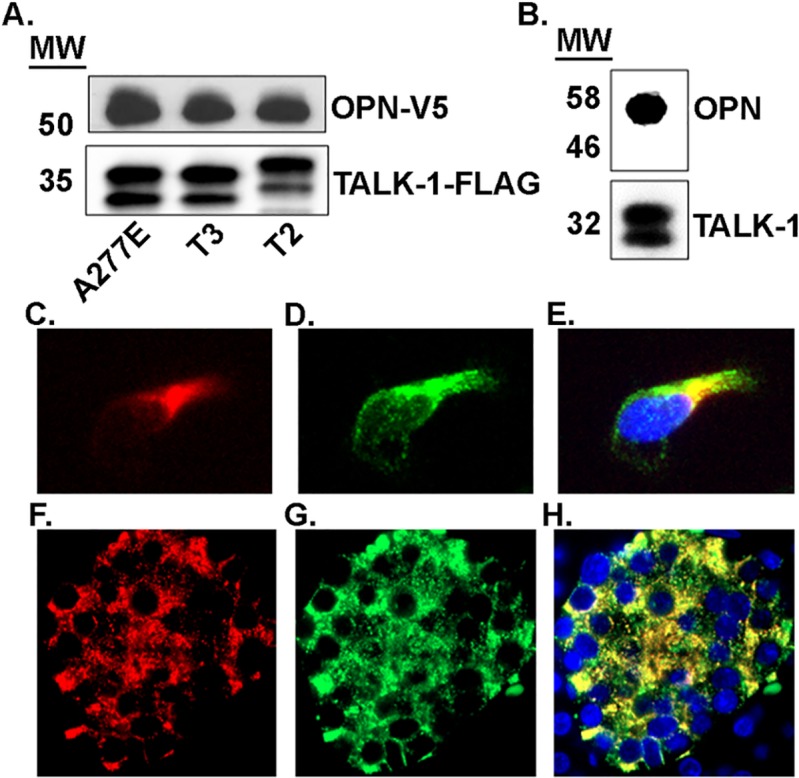
TALK-1 interacts with iOPN in a heterologous expression system (HEK293) and in primary mouse islets. A. Western blot run with TALK-1-FLAG immune complexes (isolated from HEK293 cells expressing iOPN-V5 and TALK-1-FLAG) probed with anti-V5. B. Western blot run with TALK-1 immune complexes (isolated from primary human islets) probed with anti-OPN. Immunofluorescent image of a HEK293 cell coexpressing C. OPN-V5 (red) and D. TALK-1-FLAG (green). E. Overlay of C. (OPN-V5) and D. (TALK-1-FLAG) demonstrating colocalization when heterologously expressed in HEK293 cells. Immunofluorescent image of a human pancreas section with F. endogenous OPN (red) and G. endogenous TALK-1 (green). H. Overlay of F. (OPN) and G. (TALK-1) showing islet specific colocalization (yellow).

Coimmunoprecipitation with TALK-1 T3 was used to verify protein-protein interactions in a human cell line (HEK293). TALK-1 T3 successfully pulled down iOPN (55 kDa, Fig 1A), TMX2 (34 kDa, S1A Fig), and CLDN10b (25 kDa, S1D Fig) when heterologously expressed in HEK293 cells (Fig 1A). The interaction of iOPN was further characterized with TALK-1 T2 and a diabetes-associated variant of T3 (T3 A277E). All TALK-1 variants (~35 kDa including the FLAG tag) coimmunoprecipitated iOPN. Moreover, an interaction of primary islet TALK-1 channels (~32 kDa) with iOPN (55 kDa) was confirmed in human islets with coimmunoprecipitation (Fig 1B). This confirms that the interactions identified in the MYTH assay also occur in human islets.

Next, immunofluorescence was used to investigate the cellular localization of heterologously expressed TALK-1 and iOPN in HEK293 cells as well as endogenous TALK-1 channels and iOPN in primary human islets. Substantial colocalization of TALK-1 and iOPN was observed when the proteins were heterologously expressed (tM1 = 0.825 and tM2 = 0.991; Fig 1C–1E) as well as in primary human islets (tM1 = 0.635 and tM2 = 0.877; Fig 1F–1H). Additionally, both TALK-1 and iOPN staining was highly specific to islets with no staining observed in the surrounding acinar tissue indicating that this interaction occurs predominantly in pancreatic islets.

### Identification of iOPN as an activator of TALK-1 K^+^ flux

**Fig 2:**
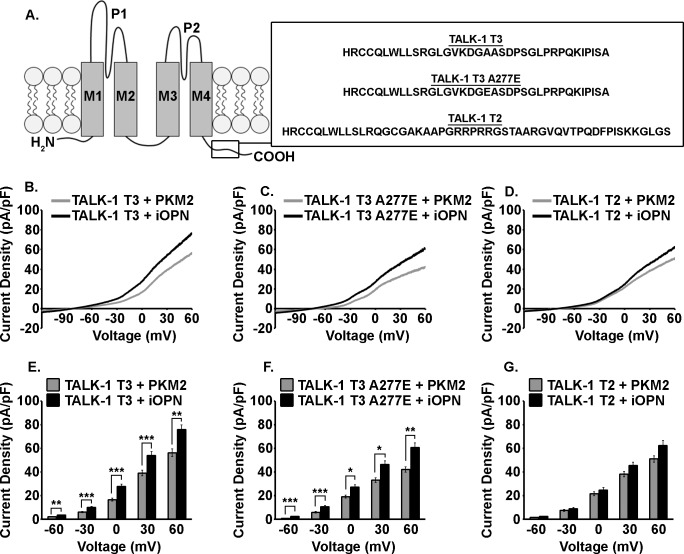
iOPN activates TALK-1 K^+^ channel currents in a TALK-1 transcript variant dependent manner. A. Illustration of the TALK-1 K2P channel and an overview of the differences in the C-terminal tails of the TALK-1 transcript variants tested. B. Average voltage-clamp recordings of K2P currents in T-REx cell stably expressing TALK-1 T3 and transfected with PKM2 (gray) or iOPN (black), C. average voltage-clamp recordings of K2P currents in T-REx cell stably expressing TALK-1 T3 A277E and transfected with PKM2 (gray) or iOPN (black), and D. average voltage-clamp recordings of K2P currents in T-REx cell stably expressing TALK-1 T2 and transfected with PKM2 (gray) or iOPN (black). For these recordings the command voltage was first held at -80 mV for 15 sec then followed with a voltage ramp from -120 mV to +60 mV (ramp duration = 1 sec). E. Quantification of K2P current densities at -60, -30, 0, 30, and 60 mV for T-Rex cells stably expressing TALK-1 T3 and transfected with PKM2 (gray) or iOPN (black), F. quantification of K2P current densities at -60, -30, 0, 30, and 60 mV for T-Rex cells stably expressing TALK-1 T3 A277E and transfected with PKM2 (gray) or iOPN (black), and G. quantification of K2P current densities at -60, -30, 0, 30, and 60 mV for T-Rex cells stably expressing TALK-1 T2 and transfected with PKM2 (gray) or iOPN (black). Data are a mean of N ≥ 10 with uncertainty expressed as SEM (*P < 0.05, **P < 0.01, ***P < 0.001).

Modulation of TALK-1 K^+^ flux, measured using a whole cell patch clamp technique, was employed to quantify the effect of identified protein interactions on TALK-1 channel activity. The interacting proteins displayed divergent effects on TALK-1 function either activating, inhibiting, or displaying no effect TALK-1 channel currents. For example, TMX2 had no effect on TALK-1 channel function (S1C and S1D Fig) while CLDN10b showed modest activation of TALK-1 channel activity (S1E and S1F Fig). Of the TALK-1-interacting proteins that were analyzed, iOPN, a potent TALK-1 activator, was selected for further investigation (Fig 2).

Intracellular OPN or a non-interacting control (PKM2) was expressed in stable cell lines that allow tetracycline induced expression of TALK-1 variants (T2, T3, and T3 A277E). Intracellular OPN expression significantly increased TALK-1 T3 currents over the range of *V*_m_ from -60 to +60 mV compared to control (1.3 ± 0.4, 4.0 ± 1.0, 11.0 ± 2.5, 14.9 ± 4.1, and 19.6 ± 5.2, P < 0.01, Fig 2B and 2E). Similarly, iOPN expression increased TALK-1 T3-A277E currents compared to control over the same range from -60 to +60 mV (1.6 ± 0.4, 4.7 ±1.3, 8.1 ± 2.3, 13.3 ± 3.7, and 18.7 ± 4.5 pA/pF, P < 0.05, Fig 2C and 2F). Although iOPN expression strongly activated TALK-1 T3 channels, it only minimally activated TALK-1 T2 currents at non-physiological voltages (Fig 2D and 2G). These results indicate that the C-terminal tail of TALK-1 is a critical determinant of the magnitude of TALK-1 channel activation by iOPN, whereas, the N-terminus of TALK-1 is important for the interaction with iOPN.

### TALK-1 K^+^ currents are activated by iOPN in primary mouse β-cells

**Fig 3:**
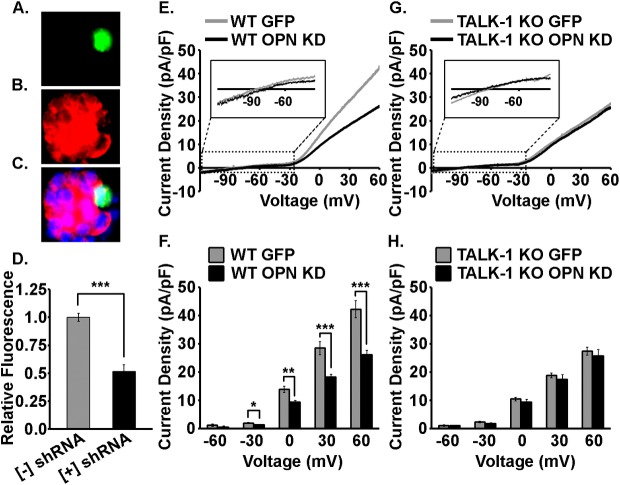
iOPN activates TALK-1 K^+^ currents in primary mouse β-cells. A. An OPN shRNA transfected cell that also expresses EGFP for identification (green), B. islet localized iOPN immunofluorescence (red), and C. a merged image showing iOPN KD in a transfected cell. D. Quantification of iOPN KD based on OPN immunofluorescence in EGFP positive and EGFP negative β-cells. Data are a mean of N ≥ 13 cells with uncertainty expressed as SEM. (***P < 0.001). E. Average voltage-clamp recordings of K2P currents in WT mouse β-cells expressing EGFP alone (gray) or OPN shRNA/EGFP (black). F. K2P current densities quantified at -60, -30, 0, 30, and 60 mV for WT mouse β-cells expressing EGFP alone (gray) or OPN shRNA/EGFP (black). G. Average voltage-clamp recordings of K2P currents in TALK-1 KO mouse β-cells expressing EGFP alone (gray) or OPN shRNA/EGFP (black). H. K2P current densities quantified at -60, -30, 0, 30, and 60 mV for TALK-1 KO mouse β-cells expressing EGFP alone (gray) or OPN shRNA/EGFP (black). For these recordings the command voltage was first held at -80 mV for 15 sec then followed with a voltage ramp from -120 mV to +60 mV (ramp duration = 1 sec). Insets show expansions of the regions around the reversal potentials of K2P channel recordings. Data are a mean of N ≥ 10 cells from three animals with uncertainty expressed as SEM. (*P < 0.05, **P < 0.01, ***P < 0.001).

Next, the impact of endogenous iOPN on TALK-1 K^+^ channel function was evaluated in primary mouse β-cells in response to a voltage ramp. Intracellular OPN was knocked down in WT and TALK-1 KO mouse islet clusters using an shRNA-based approach (Fig 3A–3C; Note: EGFP was also expressed in all cells expressing the OPN shRNA) and β-cell K2P currents were measured from EGFP positive cells. Expression of OPN shRNA reduced β-cell iOPN by at least 48.4 ± 5.2% based on β-cell immunofluorescence analysis (Fig 3D). Due to modest background fluorescence it is likely that the OPN knockdown efficiency was actually higher. Knockdown of iOPN in WT islet clusters significantly reduced β-cell K2P currents in the -30 to +60 mV voltage range (0.6 ± 0.2, 4.5 ± 1.3, 10.2 ± 2.6, and 16.1 ± 3.4 pA/pF, P < 0.05; Fig 3E and 3F). Furthermore, KD of iOPN in TALK-1 KO islet clusters had no significant effect on β-cell K2P currents indicating that iOPN activation of β-cell K2P currents is only due to activation of TALK-1 channels (Fig 3G and 3H). These results strongly suggest that β-cell iOPN activates TALK-1 channels.

### Activation of TALK-1 channels by iOPN leads to *V*_m_ hyperpolarization

**Fig 4:**
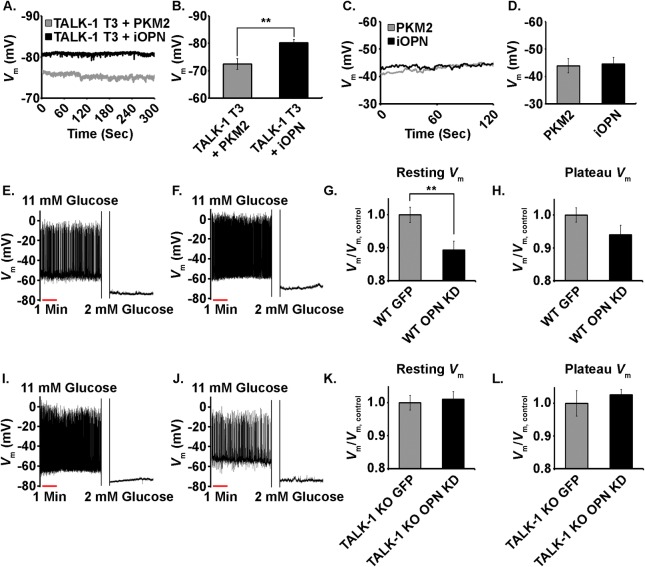
Activation of TALK-1 by iOPN hyperpolarizes the *V*_m_ of human cells (HEK293) and primary mouse β-cells. A. Representative resting *V*_m_ for heterologously expressed TALK-1 T3 and PKM2 (gray) as well as TALK-1 T3 and iOPN (black) in T-REx cells recorded using a perforated-patch technique. B. Quantification of the *V*_m_ of T-Rex cells expressing TALK-1 T3 and PKM2 (gray) or TALK-1 T3 and iOPN (black). Data are a mean of N = 10 with uncertainty expressed as SEM (**P < 0.01). C. Representative *V*_m_ recordings for HEK293 cells expressing PKM2 (gray) or iOPN (black). D. Quantification of the *V*_m_ of HEK293 cells expressing PKM2 (gray) or iOPN (black). Data are a mean of N ≥ 6. E. *V*_m_ recording of a WT mouse β-cell expressing EGFP alone and F. *V*_m_ recording of a WT mouse β-cell expressing OPN shRNA/EGFP. G. Quantification of resting *V*_m_ for WT mouse β-cells expressing EGFP alone (gray) or OPN shRNA/EGFP (black). H. Quantification of plateau *V*_m_ for WT mouse β-cells expressing EGFP alone (gray) or OPN shRNA/EGFP (black). I. *V*_m_ recording of a TALK-1 KO mouse β-cell expressing EGFP alone and J. *V*_m_ recording of a TALK-1 KO mouse β-cell expressing OPN shRNA/EGFP. K. Quantification of resting *V*_m_ for TALK-1 KO mouse β-cells expressing EGFP alone (gray) or OPN shRNA/EGFP (black). G. Quantification of plateau *V*_m_ for TALK-1 KO mouse β-cells expressing EGFP alone (gray) or OPN shRNA/EGFP (black). Data are a mean of N ≥ 8 with uncertainty expressed as SEM (**P < 0.01).

Because TALK-1 channels are constitutively active, when *V*_m_ is greater than the equilibrium potential of K^+^ (~-89 mV) activation of TALK-1 by iOPN would be expected to lead to a hyperpolarization in resting *V*_m_. Indeed, expression of TALK-1 T3 with iOPN (*V*_m_ = -80.2 ± 1.2 mV) led to a significant hyperpolarization of *V*_m_ when compared to TALK-1 T3 with a non-interacting control (PKM2) (*V*_m_ = -72.5 ± 1.9 mV, Δ*V*_m_ = -7.7 ± 2.3 mV, P < 0.01, Fig 4A and 4B). Moreover, the observed *V*_m_ hyperpolarization was due specifically to an interaction of iOPN with TALK-1 channels as the *V*_m_ of HEK293 cells expressing iOPN alone (*V*_m_ = -44.5 ± 2.5 mV) was indistinguishable from cells expressing PKM2 (*V*_m_ = -43.9 ± 2.6 mV, Fig 4C and 4D), both of which were in the normal range for untransfected HEK293 cells. This suggests that iOPN could regulate β-cell *V*_m_ and Ca^2+^ influx through VDCCs.

To demonstrate that *V*_m_ hyperpolarization due to iOPN activation of TALK-1 channels was not an consequence of using a heterologous system OPN was knocked down using an shRNA-based approach in primary β-cells then resting *V*_m_ and plateau *V*_m_ were measured under low (2.0 mM) and high (11.0 mM) glucose conditions respectively. Changes in *V*_m_ due to OPN KD were normalized to EGFP control β-cells. Thus, a decrease relative to control indicates *V*_m_ depolarization while an increase represents *V*_m_ hyperpolarization. Knockdown of iOPN in WT β-cells led to significant depolarization of resting *V*_m_ (Δ*V*_m_ = - 0.11 ± 0.04, P < 0.05, Fig 4E–4H). There was no significant difference in the plateau *V*_m_ (Δ*V*_m_ = -0.06 ± 0.04). Both resting *V*_m_ and plateau *V*_m_ were indistinguishable when iOPN was knocked down in TALK-1 KO β-cells (Δ*V*_m_ = 0.01 ± 0.03 and 0.03 ± 0.04 respectively, Fig 4I–4L). These results reinforce the ability of iOPN to activate β-cell TALK-1 channels resulting in plasma membrane hyperpolarization.

### Activation of TALK-1 channels by iOPN increases glucose-stimulated Ca^2+^ influx into primary mouse β-cells

**Fig 5:**
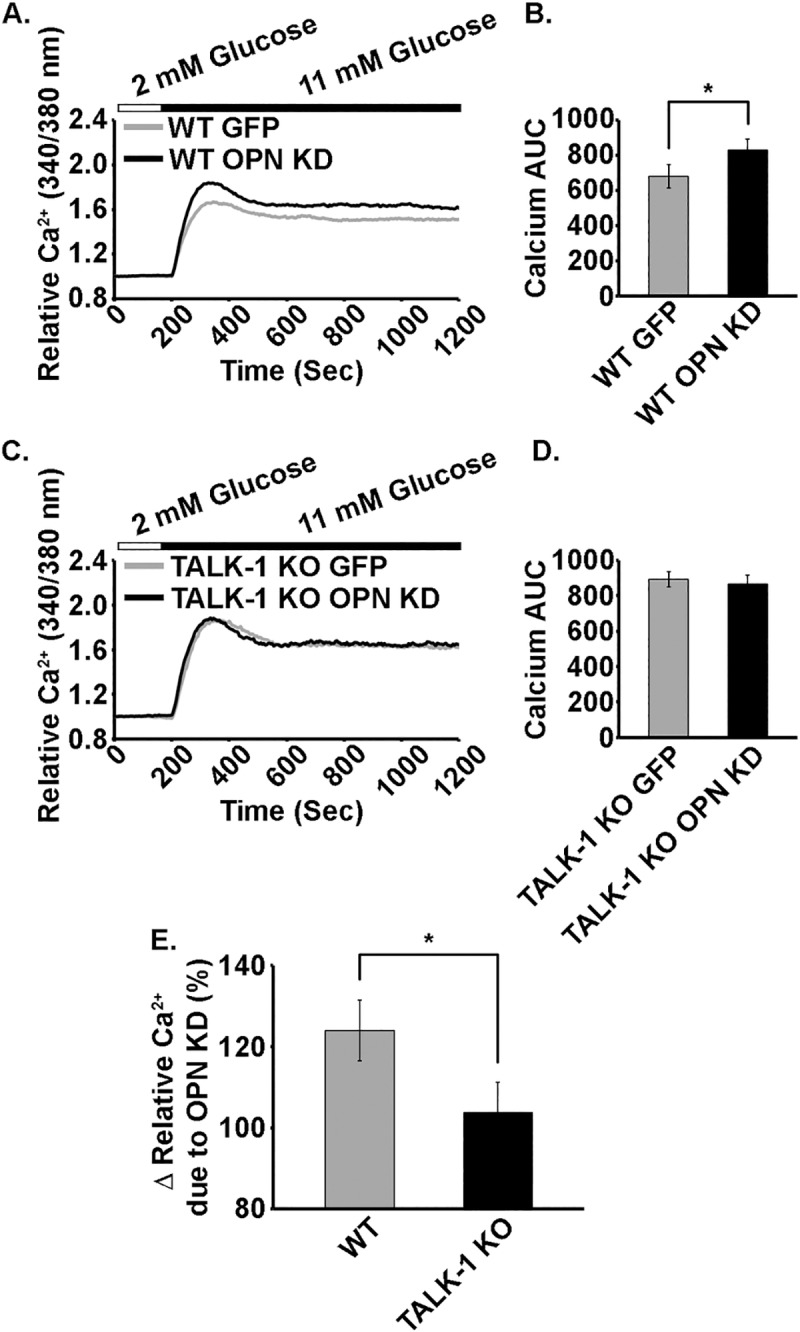
The activation of TALK-1 by iOPN limits glucose-stimulated Ca^2+^ influx into mouse β-cells. A. Glucose-stimulated Ca^2+^ influx into WT mouse β-cells monitored with the fura-2 AM Ca^2+^ dye in cells with EGFP (gray) or with OPN shRNA/EGFP (black) (the traces are the averages of all beta-cell recordings). B. Quantification of the glucose-stimulated area under the curve (AUC) (from 200–1200 sec) for WT mouse β-cells with EGFP (gray) or with OPN shRNA/EGFP (black). C. Ca^2+^ influx in TALK-1 KO mouse β-cells with EGFP (gray) or with OPN shRNA/EGFP (black) following glucose-stimulation as indicated above the traces. D. Quantification of the glucose-stimulated AUC for TALK-1 KO mouse β-cells with EGFP (gray) or with OPN shRNA/EGFP (black). E. Percent change in glucose-stimulated Ca^2+^ influx into WT and TALK-1 KO mouse β-cells following OPN KD. Data are a mean of N ≥ 41 cells for each group from five animals with uncertainty expressed as SEM. (*P < 0.05).

As TALK-1 channels function to limit β-cell VDCC activity through hyperpolarization of *V*_m_, loss of iOPN, which activates the channel, would be expected to increase β-cell glucose-stimulated Ca^2+^ influx. In order to test this, iOPN was knocked down in WT and TALK-1 KO islet clusters composed of 10–20 cells and Ca^2+^ levels were monitored. Ca^2+^ influx in response to glucose stimulation (11 mM) was significantly greater in WT β-cells with physiological levels of iOPN compared to those with iOPN KD (Fig 5A). Moreover, the glucose-stimulated area under the curve (AUC) increased significantly when iOPN was knocked down (830.2 arbitrary units (AU)) compared to EGFP controls (681.2 ± 67.5 AU; average of ≥ 51 cells per group with N = 5 mice, P < 0.05, Fig 5B). In contrast, there was no difference in β-cell glucose-stimulated Ca^2+^ influx between TALK-1 KO β-cells with physiological levels of iOPN (892.1 ± 42.5 AU) and those with iOPN knocked down (864.5 ± 52.1 AU; average of ≥ 41 cells per group with N = 5 mice; Fig 5C and 5D). The percent increase in glucose-stimulated Ca^2+^ influx following KD of iOPN was also significantly greater in WT β-cells (125.0 ± 7.7%, calculated as iOPN KD glucose-stimulated AUC /EGFP control glucose-stimulated AUC) compared to TALK-1 KO β-cells (96.7 ± 3.4%, P < 0.001; Fig 5E). These results indicate that pancreatic β-cell iOPN augments TALK-1 channel activity limiting glucose-stimulated Ca^2+^ influx.

## Discussion

TALK-1 channel modulation of β-cell *V*_m_ regulates Ca^2+^ influx and second phase GSIS [6]. A coding sequence polymorphism that results in a GOF of TALK-1 channels is also responsible for an increased risk of developing type 2 diabetes (T2D) [19, 20]. However, the mechanisms that influence beta-cell TALK-1 channel activity have not been determined. Here we utilized an unbiased MYTH assay to identify TALK-1 channel-interacting proteins from human islets, which enabled us to identify the first protein interactions with TALK-1 channels. These interacting partners of TALK-1 will be useful in identifying β-cell regulatory complexes that control TALK-1 channel activity and contribute to islet function.

Twenty-one proteins were identified that interact with TALK-1 channels. Interestingly, a large percentage of the proteins are transmembrane proteins and possess a secondary structure similar to TALK-1 (4 transmembrane domains, 2 extracellular loops, and intracellular N- and C- termini) [22, 29–31]. Many of these proteins belong to the tetraspanin family, which assemble in tetraspanin enriched microdomains (TEMs) at the plasma membrane and serve a number of important roles including protein trafficking [32–34]. They may serve a similar role for TALK-1, assisting in trafficking to the plasma membrane and possibly forming TALK-1-rich microdomains. Claudin 10b (CLDN10b), a protein important to tight junction formation and with a structure similar to tetraspanins, was also found to interact with TALK-1 [35–37]. Tetraspanins, CLDNs, and K2P channels all display one large extracellular loop and a second smaller extracellular loop [38–40]. Disulfide bonds between these extracellular loops are important to protein function and complex formation and thus the structural similarities between these proteins may enable heteromeric complex formation stabilized through disulfide linkages [38–40]. Future studies will confirm the localization and functional interactions of these tetraspanin-like proteins with TALK-1 channels.

Despite strong evidence that TALK-1 forms functional K^+^ channels at the plasma membrane (9, 11), intracellular staining was also observed in pancreas sections as well as when heterologously expressed in HEK293 cells (9). In line with this observation, our MYTH assay identified a number of intracellular proteins such as iOPN, selenoprotein K (SELK), and thioredoxin-related transmembrane protein 2 (TMX2). Furthermore, we found that TALK-1 and iOPN both exhibited similar punctate patterns, with TALK-1 appearing to be marginally more diffuse than iOPN. This may indicate localization of TALK-1 and iOPN to one or more intracellular organelles such as the endoplasmic reticulum (ER). Indeed, a recent study by Wendt et al. found that iOPN knockout in a mouse model results in reduced β-cell ER Ca^2+^ and reduction in sarco/endoplasmic reticulum Ca^2+^-ATPase 2b (SERCA2b) transcript supporting this possibility [41]. While this suggests an intracellular role for TALK-1/iOPN interactions, intracellular pools of TALK-1/iOPN complexes may also allow for transport to the plasma membrane as the channels are required. Research is ongoing to determine how intracellular TALK-1 interactions influence β-cell function independent of their role modulating *V*_m_ and how they affect trafficking of TALK-1 to the plasma membrane.

Although the roles of intracellular TALK-1 complexes remain unclear, a subset of TALK-1-interacting proteins significantly modulated K^+^ flux through plasma membrane TALK-1 channels. For example, an interaction with iOPN was found to strongly augment TALK-1 K^+^ flux. OPN is an aspartic acid-rich, Ca^2+^ binding phosphoprotein expressed in many tissues and cells as well as body fluids, which belongs to the SIBLING (small integrin-binding ligand N-linked glycoprotein) protein family [42–44]. Many forms of OPN exist including three splice variants (OPNa, OPNb and OPNc) as well as a protein variant that localizes in the cytoplasm known as intracellular OPN (iOPN) that results from alternative translation [45]. Osteopontin serves diverse roles including binding to calcium-based biominerals, such as calcium-phosphate, that is important to inhibition and regulation of bone mineralization [46]. Several physiological roles have also been identified for iOPN including cytoskeleton rearrangement, modulation of immune responses, and as an adaptor or scaffolding protein [42, 45, 47]. Similar to previous reports, we found that iOPN is highly expressed in human pancreatic islets and beta-cells [48]. While OPN stimulation of integrins can influence ion channel activity [49–52], this is the first report of a direct interaction of iOPN with an ion channel. This does not exclude the possibility that a portion of iOPN induced activation of TALK-1 channels is due to indirect signaling mechanisms. However, exogenous stimulation of rodent islets with OPN does not stimulate GSIS [42]. Therefore, iOPN plasma membrane *V*_m_ hyperpolarization and reduction of β-cell Ca^2+^ influx by activation of TALK-1 channels must occur through intracellular mechanisms, presumably through a direct interaction with TALK-1 channels. Importantly, this predicts that TALK-1/iOPN interactions serve to tune β-cell GSIS.

Intracellular OPN levels change in response to a variety of conditions, which may lead to dynamic modulation of beta-cell TALK-1 channel activity and GSIS. A wide range of inflammatory cytokines and growth factors have been shown to regulate expression of iOPN including LPS, NO, IL-1β, IFN-γ, and TNF-α [42, 43, 45, 47]. Hyperglycemic conditions and the incretin hormone glucose-dependent insulinotropic polypeptide (GIP) also increase OPN mRNA and protein content in pancreatic islets [48]. Furthermore, it has been well documented that low level inflammation associated with the early stages of T2D leads to upregulation of OPN mRNA and iOPN protein expression in pancreatic islets [42, 44, 47, 53]. Elevation of iOPN serves a protective role in pancreatic islets where it acts as a negative feedback regulator of induced NO synthase (iNOS) and as a promoter of a protective Th2 immune response [42, 44, 47, 54]. Interestingly, TALK-1 channels are activated by NO. Therefore, the reduction of NO production resulting from increased iOPN may lead to a transient reduction in TALK-1 K^+^ currents, which would be expected to increase Ca^2+^ influx and GSIS. However, iOPN itself strongly increases TALK-1 channel activity, which would likely offset indirect effects, hyperpolarize beta-cell *V*_m_, and ultimately reduce Ca^2+^ influx and GSIS. Upregulation of iOPN occurs during conditions of chronically elevated beta-cell insulin secretion. As excessive insulin secretion can result in endoplasmic reticulum (ER) stress and exacerbate beta-cell dysfunction, activation of TALK-1 channels by iOPN may serve to limit GSIS in order to reduce ER stress and apoptotic signaling [55–57]. Thus, modulation of TALK-1 activity by iOPN may contribute to the disruption in insulin secretion observed in pancreatic islets under inflammatory conditions.

Interestingly, while both K^+^ channel forming variants of TALK-1 interacted with iOPN, only TALK-1 T3 channels were activated by iOPN at physiological voltages. This suggests that although the iOPN interaction with TALK-1 channels occurs in the N-terminal domain that is conserved between all TALK-1 variants, activation of TALK-1 channels by iOPN depends on the C-terminal tail. It has been established that other K2P channels are regulated by their C-termini, which control activity by exerting either opening or closing forces on the C-type gate. For example, the C-termini of TREK-1 channels are in close proximity to the membrane when the C-type gate is open and pushed away from the membrane when the C-type gate is closed [29, 58]. Stimuli that activate TREK-1 increase the strength of the interaction between the C-terminus and the plasma membrane (e.g. acidic conditions, polyunsaturated fatty acids, and anesthetics) while conditions that decrease channel activity reduce the interaction between the C-terminus of TREK-1 and the plasma membrane (e.g. phosphorylation by PKA or PKC) [29, 58]. Charged residues in the C-terminal tail of TREK-1 also help to control channel activity by increasing or decreasing the proximity to the plasma membrane. TALK-1 channels also contain charged residues in their C-terminal tails. The composition of charged residues in the C-terminal tail of TALK-1 T2 and T3 differs significantly (TALK-1 T2- 9 positively and one negatively charged residue(s) and TALK-1 T3- 4 positively and 2 negatively charged residues). Thus, one possibility is that iOPN impacts TALK-1 C-terminal tail movement in a charge dependent manner. Further studies are required to fully characterize the mechanism responsible for iOPN activation of TALK-1.

In conclusion, iOPN interacted with TALK-1 T2 and T3 but only activated TALK-1 T3 at physiologically relevant voltages. Interaction with iOPN increased TALK-1 currents causing plasma membrane hyperpolarization and decreased glucose-stimulated Ca^2+^ influx. It is known that iOPN is upregulated under the low level inflammatory conditions present during the onset of T2D. Thus, one possible function of elevated β-cell iOPN levels may be to hyperpolarize *V*_m_ by activating TALK-1 K^+^ channels and thereby limit abnormally high insulin secretion from β-cells, which would be expected to improve β-cell health by reducing ER stress and apoptosis.

## Supporting information

S1 Fig Several of the pancreatic islet proteins identified by MYTH assay interact with TALK-1 channels and a subset of these proteins also modulate TALK-1 channel function. A. Western blot run with TALK-1 T3-FLAG immune complexes (isolated from HEK293 cells expressing thioredoxin related transmembrane protein 2 (TMX2)-V5 and TALK-1 T3-FLAG) probed with anti-V5. B. Average voltage-clamp recordings of K2P currents in cells expressing TALK-1 T3 and transfected with PKM2 (gray) or TMX2 (black). C. Quantification of K2P current densities at -60, -30, 0, 30, and 60 mV for cells expressing TALK-1 T3 and transfected with PKM2 (gray) or TMX2 (black). D. Western blot run with TALK-1 T3-FLAG immune complexes (isolated from HEK293 cells expressing members of the claudin (CLDN) family (CLDN4, CLDN7, and CLDN10)-V5 and TALK-1 T3-FLAG) probed with anti-V5. E. Average voltage-clamp recordings of K2P currents in cells expressing TALK-1 T3 and transfected with PKM2 (gray) or CLDN10 (black). F. Quantification of K2P current densities at -60, -30, 0, 30, and 60 mV for cells expressing TALK-1 T3 and transfected with PKM2 (gray) or CLDN10 (black).
(TIF)

S2 Fig The intracellular C-terminal tail of TALK-1 is not required for the channel to interact with iOPN. A. Western blot run with TALK-1 T3-FLAG immune complexes (isolated from HEK293 cells expressing OPN-V5 and TALK-1 T3-FLAG) probed with anti-V5 and anti-FLAG. B. Western blot run with TALK-1 T3-FLAG immune complexes (isolated from HEK293 cells expressing OPN-V5 and TALK-1 T3-FLAG mutant with the C-terminal tail deleted (TALK-1 T3 Δ259-294-FLAG)) probed with anti-V5 and anti-FLAG.
(TIF)

S3 Fig OPN localizes to the cytoplasm when heterologously expressed with TALK-1 channels. The cellular localization of iOPN (red) when expressed with TALK-1 channels was investigated. Nuclei (blue) were visualized with a DAPI stain. A. Representative immunofluorescent surface staining for OPN in a HEK293 cell with heterologously expressed OPN where anti-OPN was applied prior to cell fixation in order to prevent internalization B. Representative immunofluorescent staining for OPN in a fixed and permeablized HEK293 cell with heterologously expressed OPN.
(TIF)

S4 Fig Western blots demonstrating specificity of FLAG pulldown and antibodies. A. Western blot run with TALK-1 T3-FLAG immune complexes (anti-V5 or anti-FLAG) isolated from HEK293 cells showing specific pulldown with anti-FLAG. B. Western blot run with cell lysates isolated from HEK293 cells expressing OPN-V5 or TALK-1 FLAG showing specificity of anti-FLAG. C. Western blot run with cell lysates isolated from HEK293 cells expressing OPN-V5 or TALK-1 FLAG showing specificity of anti-V5. D. Western blot run with cell lysates isolated from HEK293 cells expressing OPN-V5 or TALK-1 FLAG showing specificity of anti-OPN. E. Western blot run with cell lysates isolated from tetracycline induced and uninduced T3H16 cells showing specificity of anti-TALK-1.
(TIF)
