# Supplementary figures and images for: Osteopontin activates the diabetes-associated potassium channel TALK-1 in pancreatic β-cells
Source: PLoS One. 2017 Apr 12;12(4):e0175069. doi: 10.1371/journal.pone.0175069 (PMC5389796; doi:10.1371/journal.pone.0175069)

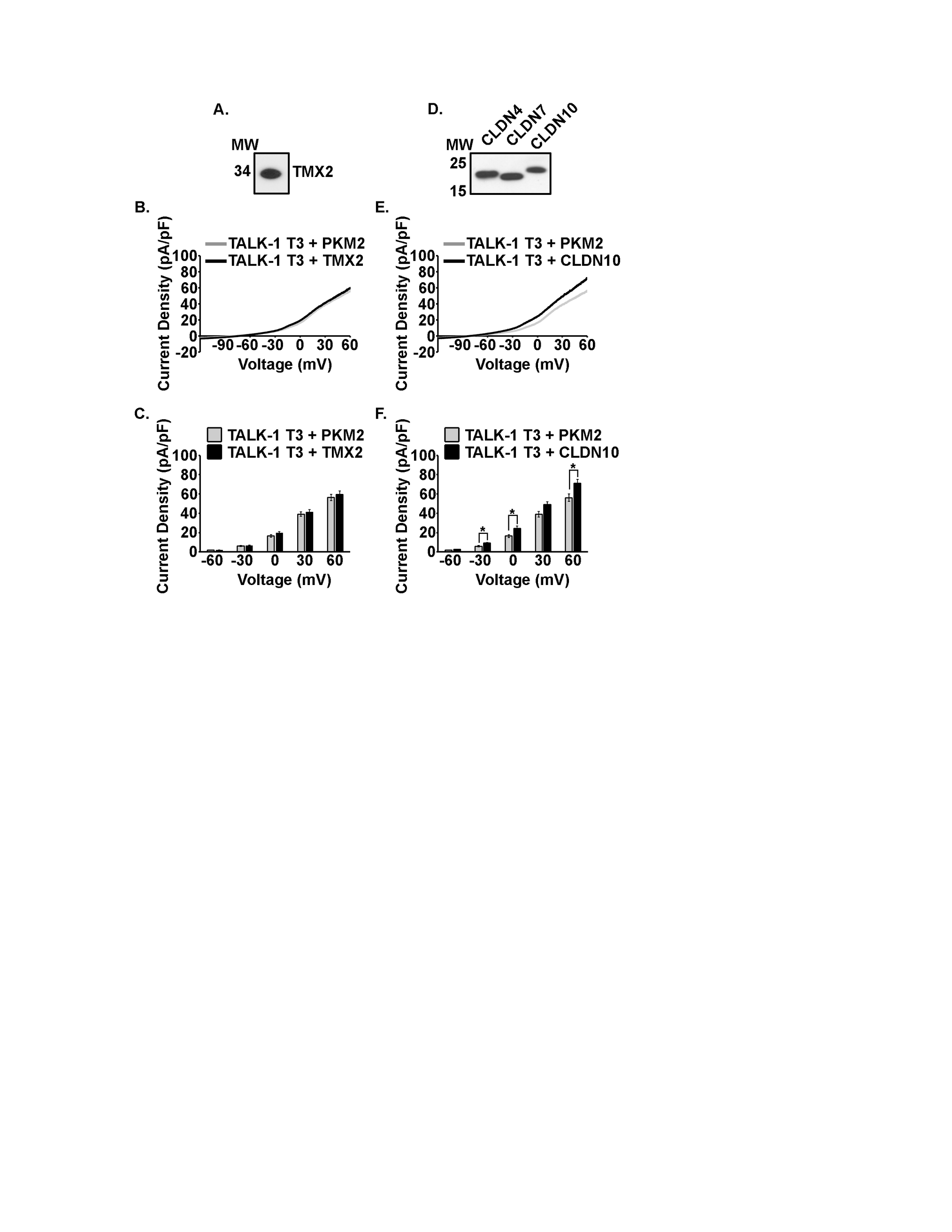

Supplement: S1 Fig — A. Western blot run with TALK-1 T3-FLAG immune complexes (isolated from HEK293 cells expressing thioredoxin related transmembrane protein 2 (TMX2)-V5 and TALK-1 T3-FLAG) probed with anti-V5. B. Average voltage-clamp recordings of K2P currents in cells expressing TALK-1 T3 and transfected with PKM2 (gray) or TMX2 (black). C. Quantification of K2P current densities at -60, -30, 0, 30, and 60 mV for cells expressing TALK-1 T3 and transfected with PKM2 (gray) or TMX2 (black). D. Western blot run with TALK-1 T3-FLAG immune complexes (isolated from HEK293 cells expressing members of the claudin (CLDN) family (CLDN4, CLDN7, and CLDN10)-V5 and TALK-1 T3-FLAG) probed with anti-V5. E. Average voltage-clamp recordings of K2P currents in cells expressing TALK-1 T3 and transfected with PKM2 (gray) or CLDN10 (black). F. Quantification of K2P current densities at -60, -30, 0, 30, and 60 mV for cells expressing TALK-1 T3 and transfected with PKM2 (gray) or CLDN10 (black). (TIF) [file pone.0175069.s001.tif]

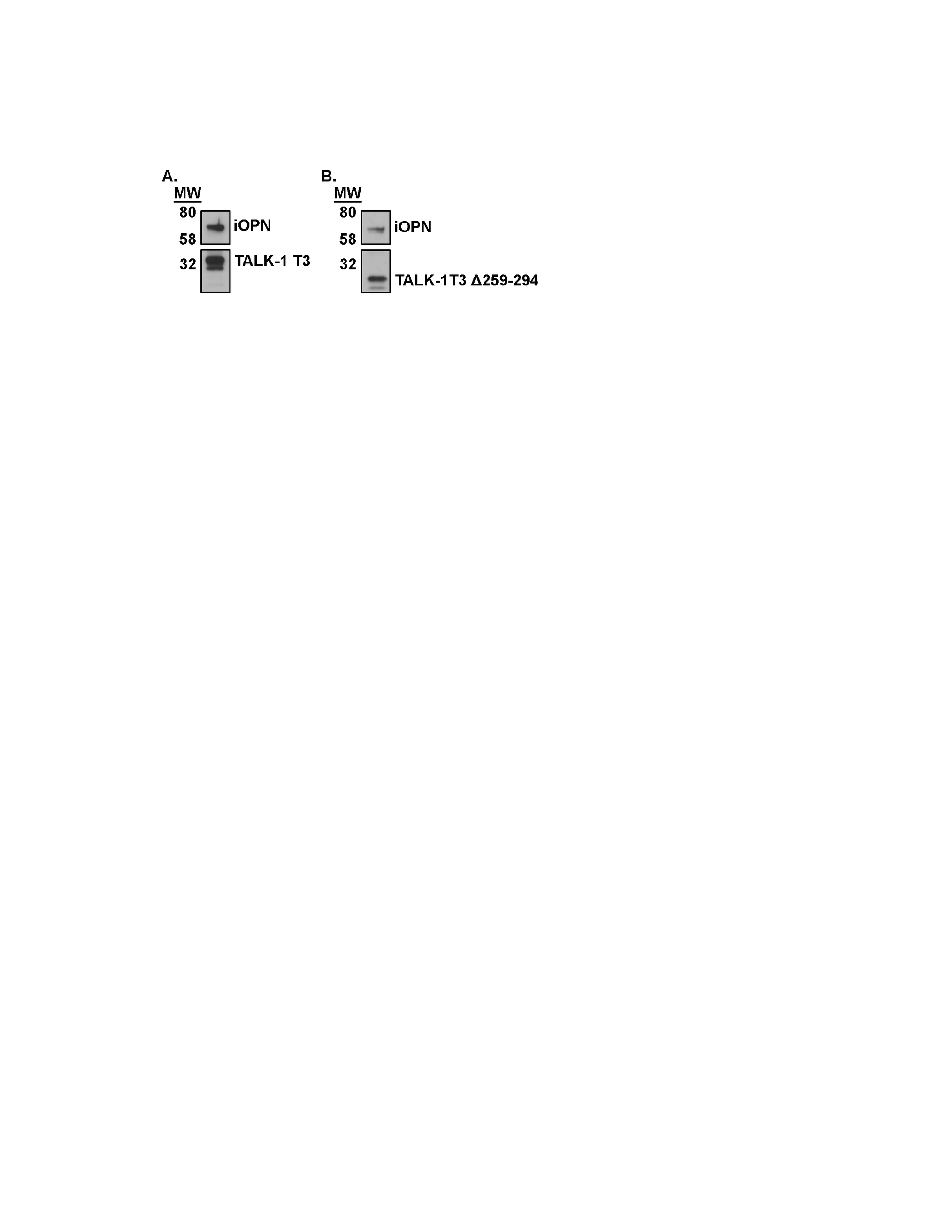

Supplement: S2 Fig — A. Western blot run with TALK-1 T3-FLAG immune complexes (isolated from HEK293 cells expressing OPN-V5 and TALK-1 T3-FLAG) probed with anti-V5 and anti-FLAG. B. Western blot run with TALK-1 T3-FLAG immune complexes (isolated from HEK293 cells expressing OPN-V5 and TALK-1 T3-FLAG mutant with the C-terminal tail deleted (TALK-1 T3 Δ259-294-FLAG)) probed with anti-V5 and anti-FLAG. (TIF) [file pone.0175069.s002.tif]

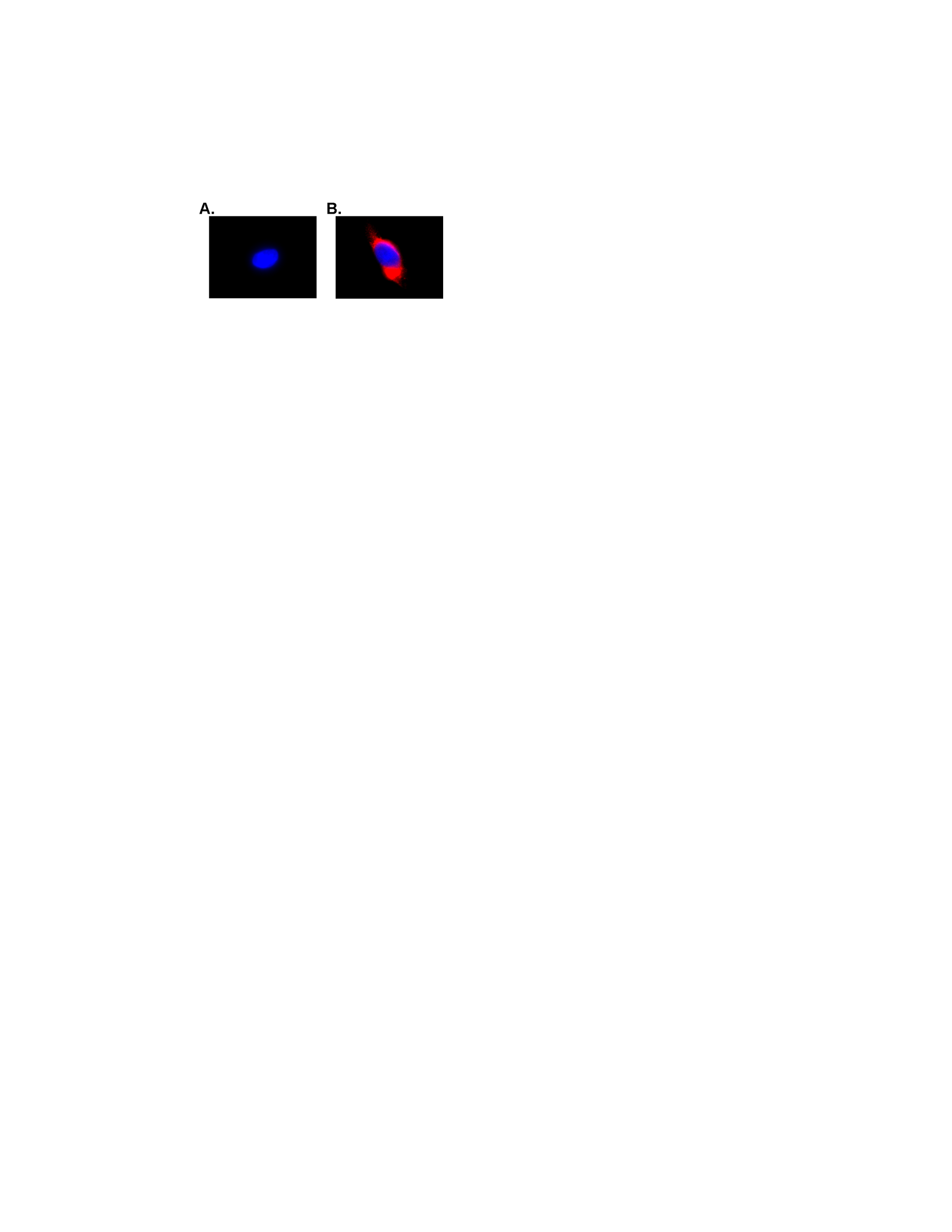

Supplement: S3 Fig — The cellular localization of iOPN (red) when expressed with TALK-1 channels was investigated. Nuclei (blue) were visualized with a DAPI stain. A. Representative immunofluorescent surface staining for OPN in a HEK293 cell with heterologously expressed OPN where anti-OPN was applied prior to cell fixation in order to prevent internalization B. Representative immunofluorescent staining for OPN in a fixed and permeablized HEK293 cell with heterologously expressed OPN. (TIF) [file pone.0175069.s003.tif]

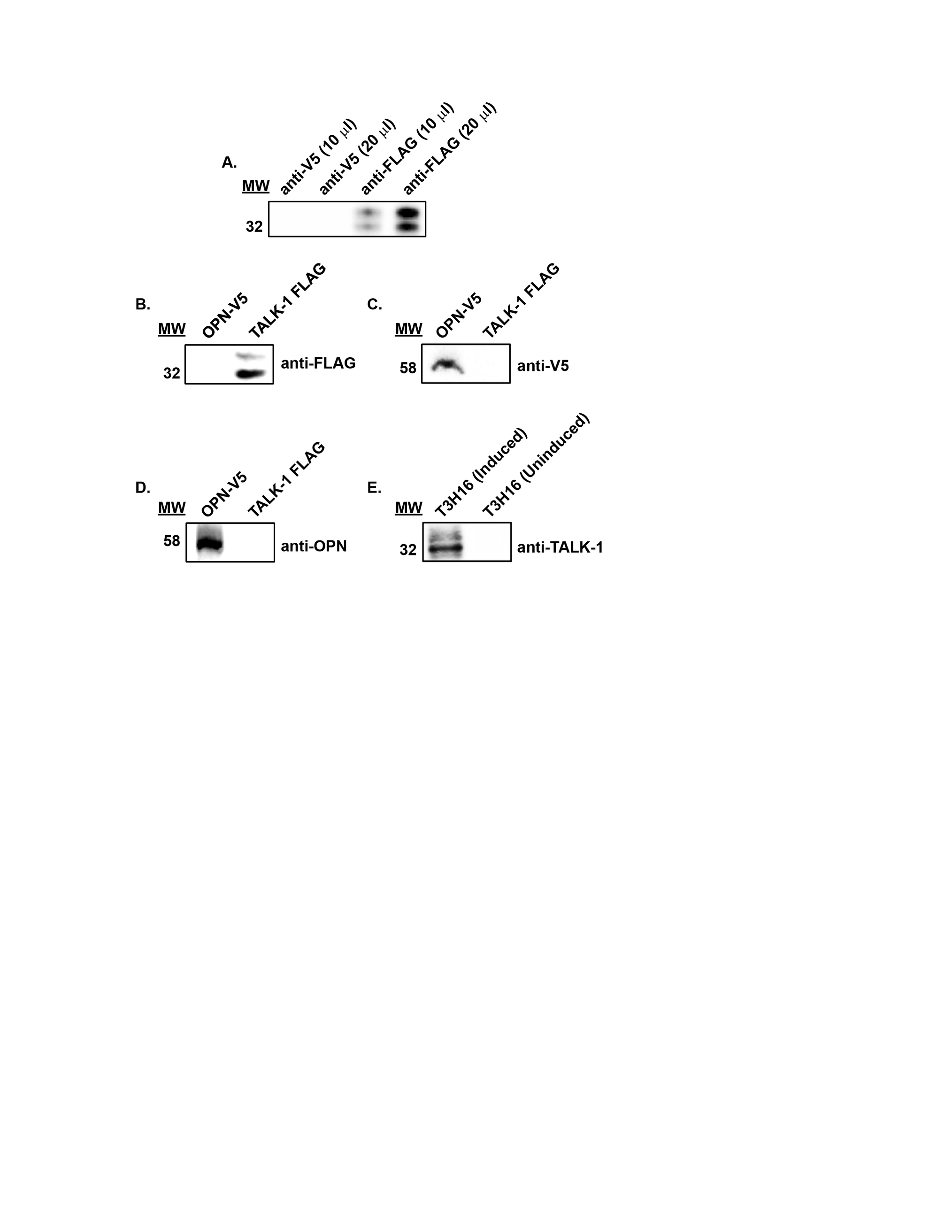

Supplement: S4 Fig — A. Western blot run with TALK-1 T3-FLAG immune complexes (anti-V5 or anti-FLAG) isolated from HEK293 cells showing specific pulldown with anti-FLAG. B. Western blot run with cell lysates isolated from HEK293 cells expressing OPN-V5 or TALK-1 FLAG showing specificity of anti-FLAG. C. Western blot run with cell lysates isolated from HEK293 cells expressing OPN-V5 or TALK-1 FLAG showing specificity of anti-V5. D. Western blot run with cell lysates isolated from HEK293 cells expressing OPN-V5 or TALK-1 FLAG showing specificity of anti-OPN. E. Western blot run with cell lysates isolated from tetracycline induced and uninduced T3H16 cells showing specificity of anti-TALK-1. (TIF) [file pone.0175069.s004.tif]
